# Mitophagy associated self-degradation of phosphorylated MAP4 guarantees the migration and proliferation responses of keratinocytes to hypoxia

**DOI:** 10.1038/s41420-023-01465-3

**Published:** 2023-05-17

**Authors:** Yanhai Feng, Lingfei Li, Qiong Zhang, Yongqing He, Yao Huang, Junhui Zhang, Dongxia Zhang, Yuesheng Huang, Xia Lei, Jiongyu Hu, Gaoxing Luo

**Affiliations:** 1 Institute of Burn Research, Southwest Hospital, Third Military Medical University (Army Medical University), Chongqing, China; 2 State Key Laboratory of Trauma, Burns and Combined Injury, Southwest Hospital, Third Military Medical University (Army Medical University), Chongqing, China; 3 Army 953 Hospital, Shigatse Branch of Xinqiao Hospital, Third Military Medical University (Army Medical University), Shigatse, China; 4 Department of Dermatology, Daping Hospital, Third Military Medical University (Army Medical University), Chongqing, China; 5 Department of Geriatric Oncology, Department of Palliative care, Department of Clinical nutrition, Chongqing University Cancer Hospital, Chongqing, China; 6 Department of Wound Repair, Institute of Wound Repair and Regeneration Medicine, Southern University of Science and Technology Hospital, Southern University of Science and Technology School of Medicine, Shenzhen, China; 7 Endocrinology Department, Southwest Hospital, Third Military Medical University (Army Medical University), Chongqing, China

**Keywords:** Cell migration, Mitophagy

## Abstract

Our previous study has announced that phosphorylated microtubule-associated protein 4 (p-MAP4) accelerated keratinocytes migration and proliferation under hypoxia through depolymerizing microtubules. However, p-MAP4 should exhibit inhibitory effects on wound healing, for it also impaired mitochondria. Thus, figuring out the outcome of p-MAP4 after it impaired mitochondria and how the outcome influenced wound healing were far-reaching significance. Herein, the results revealed that p-MAP4 might undergo self-degradation through autophagy in hypoxic keratinocytes. Next, p-MAP4 activated mitophagy which was unobstructed and was also the principal pathway of its self-degradation triggered by hypoxia. Moreover, both Bcl-2 homology 3 (BH3) and LC3 interacting region (LIR) domains had been verified in MAP4, and they endowed MAP4 with the capability to synchronously function as a mitophagy initiator and a mitophagy substrate receptor. And, mutating any one of them ruined hypoxia-induced self-degradation of p-MAP4, resulting in destroyed proliferation and migration responses of keratinocytes to hypoxia. Our findings unviewed that p-MAP4 experienced mitophagy-associated self-degradation through utilizing its BH3 and LIR domains under hypoxia. As a result, the mitophagy-associated self-degradation of p-MAP4 guaranteed the migration and proliferation responses of keratinocytes to hypoxia. Together, this research provided a bran-new pattern of proteins in regulating wound healing, and offered a new direction for intervening wound healing.

## Introduction

As an essential component of the cytoskeleton, the microtubule is rising as an important structural and functional regulator of wound healing [1]. Microtubule-associated protein 4 (MAP4) is one critical protein regulating microtubule homeostasis, and our previous research has verified that MAP4 promoted hypoxia-induced wound healing through depolymerizing microtubules in a phosphorylation-dependent manner [2]. However, the basic biological functions of phosphorylated MAP4 (p-MAP4) are mainly divided into two aspects. In detail, p-MAP4 induced microtubule depolymerization after dissociating from microtubule in hypoxia cells, and the dissociated p-MAP4 then translocated to the mitochondrial outer membrane and impaired mitochondria [3, 4]. Therefore, p-MAP4 should possess the competency to delay wound healing from the aspect of mitochondrial impairment. Contrary to this inference, p-MAP4 promotes wound healing [2]. Thus, the key problem is what is the outcome of p-MAP4 after it impairs mitochondria and how this outcome influences wound healing.

Keratinocyte autophagy has been well-identified to promote wound healing [5]. However, investigators draw this conclusion mainly based on the intervention of key molecules in the autophagy process. In addition, the autophagy-lysosomal system is also a classic way to clear damaged organelles, toxic proteins, etc., to maintain intracellular homeostasis [6]. Therefore, exploring the effects of autophagy on wound healing from the perspective of its scavenging ability is far-reaching significance. Mitophagy, a selective autophagy, is widely known for its ability to clear damaged mitochondria [7]. When mitophagy scavenges damaged mitochondria, the mitochondrial proteins and mitophagy substrate receptors are also degraded theoretically. Many proteins, such as BNIP3, have been reported to both work as autophagy activators and autophagy substrate receptors, indicating these proteins should be capable of undergoing self-degradation through mitophagy [8]. Moreover, BNIP3 has been announced to promote wound healing under hypoxia [9]. These facts raise our interests in investigating whether p-MAP4 suffered mitophagy-associated self-degradation in hypoxic keratinocytes and how this outcome impacts wound healing.

Herein, the present study confirmed that p-MAP4 experienced mitophagy-associated self-degradation in keratinocytes, and the process was triggered by hypoxia. In addition, the BH3 and LIR domains made MAP4 synchronously function as a mitophagy initiator and a mitophagy substrate receptor, and were the core reasons for its self-degradation. As a result, the self-degradation of p-MAP4 assured the proliferation and migration responses of keratinocytes to hypoxia. Thus, our results hinted that the self-degradation of p-MAP4 provided a cellular basis for hypoxia-induced wound healing. Together, this research provided a bran-new pattern of proteins in regulating wound healing, and proved that the scavenging ability of autophagy might play a more central role than its other functions in mediating wound healing.

## Results

### The self-degradation of p-MAP4 was triggered by hypoxia

**Fig. 1:**
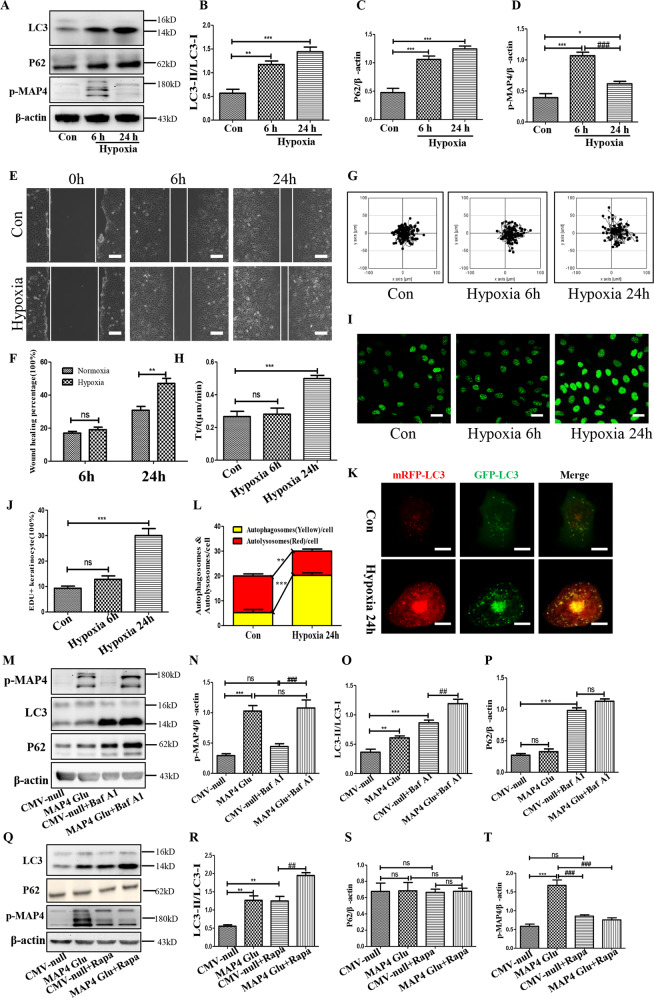
The self-degradation of p-MAP4 was triggered by hypoxia. **A**–**D** Representative bands and corresponding quantifications of LC3, P62, and p-MAP4 in hypoxic keratinocytes. *p < 0.05, **p < 0.01, and ***p < 0.001 versus Con group. *n* = 5. **E**, **F** Representative images and corresponding quantifications of scratch wound healing assays were shown. Scale bar = 100 μm. **p < 0.01 versus Con group. *n* = 5. **G**, **H** Representative images and corresponding quantifications of single-cell motility assays. ***p < 0.001 versus Con group. *n* = 5. **I**, **J** Representative images and corresponding quantifications of EdU staining (Green) in hypoxic keratinocytes. Scale bar = 50 μm. ***p < 0.001 versus Con group. *n* = 5. **K**, **L** Representative images and corresponding quantifications of autophagosome and autolysosome in keratinocytes transfected with mRFP-GFP-LC3. Scale bar = 10 μm. **p < 0.01 and ***p < 0.001 versus Con group. *n* = 5. **M**–**P** Representative bands and corresponding quantifications of LC3, P62, and p-MAP4 in keratinocytes treated by MAP4 (Glu) adenovirus with or without 10 nM Baf A1 for 1 h. **p < 0.01 and ***p < 0.001 versus Con group. ^##^*p* < 0.01 and ^###^*p* < 0.001 versus CMV-null+Baf A1. *n* = 5. **Q**–**T** Representative bands and corresponding quantification of LC3, P62, and p-MAP4 in keratinocytes treated by MAP4 (Glu) adenovirus with or without 100 nM Rapa for 24 h. **p < 0.01 and ***p < 0.001 versus Con group. ^###^*p* < 0.001 versus MAP4 (Glu). *n* = 5.

To figure out the outcome of p-MAP4 under hypoxia, its variation tendency with time was first investigated. As shown in Fig. 1A–D, p-MAP4 peaked at 6 h and decreased at 24 h, which was accompanied by the progressively elevated autophagy level (*p* < 0.05). Meanwhile, the keratinocytes migration and proliferation increased mainly at 24 h after hypoxia treatment (Fig. 1E–J, *p* < 0.05). Based on these data, and considering the autophagy-lysosomal system was one of the main ways to degrade proteins, we preliminarily speculated that p-MAP4 might be degraded by autophagy which played material roles in regulating keratinocytes migration and proliferation under hypoxia. However, p-MAP4 could not be completely degraded by hypoxia because the autophagy flux induced by hypoxia was obstructed, which was reflected by augmented p62 expressions and accumulated autophagosomes (Fig.1A, K, L, *p* < 0.05). More directly, 10 nM Bafilomycin A1 (Baf A1), which was a classical inhibitor of the fusion of lysosome and autophagosome, was utilized to further clarify the effects of obstructed autophagy flux in degrading p-MAP4, and the results demonstrated that obstructed autophagy flux could not degrade p-MAP4 (Fig.1M–P, *p* < 0.05). Next, to further confirm whether autophagy could degrade p-MAP4, 100 nM rapamycin (Rapa), an autophagy activator, was employed. And, the data demonstrated that unobstructed autophagy induced by Rapa could degrade p-MAP4 (Fig.1Q–T, *p* < 0.05). Interestingly, during the process, the activating effects of p-MAP4 on autophagy, which was reflected by increased LC3-II/LC3-I ratio, was preliminarily discovered (Fig.1M–S, *p* < 0.05). Based on these facts, we shared a bold speculation that hypoxia might trigger the autophagy-associated self-degradation of p-MAP4.

### Mitophagy played a crucial role in the hypoxia-induced self-degradation of p-MAP4

**Fig. 2:**
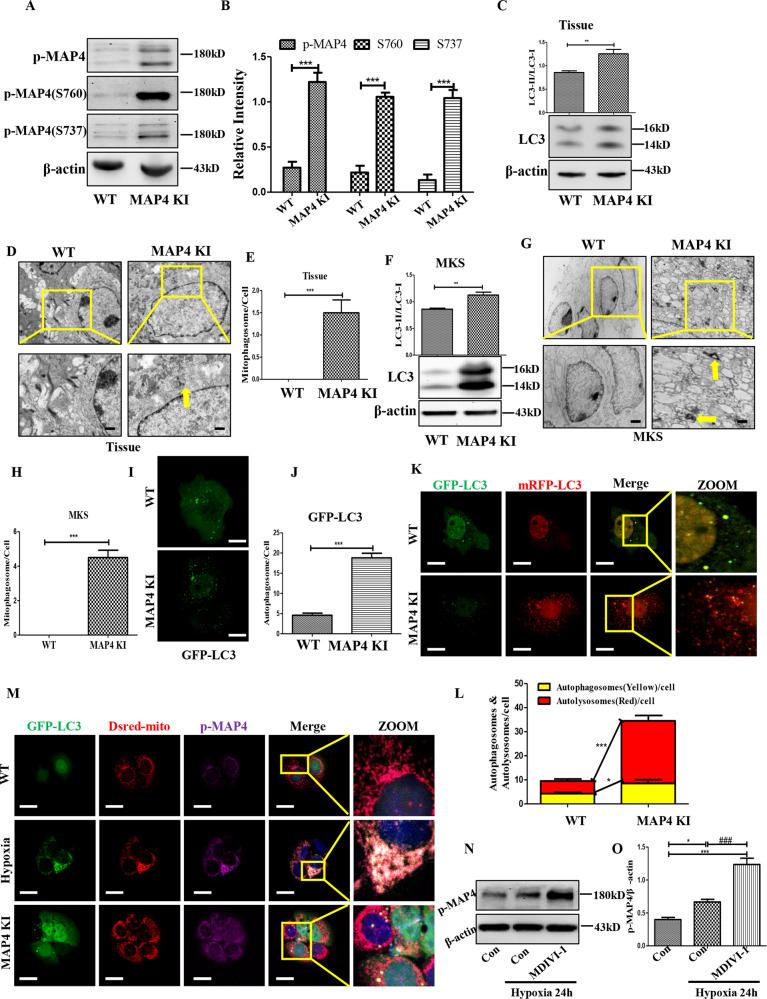
Mitophagy played a crucial role in hypoxia-induced self-degradation of p-MAP4. **A**, **B** Representative bands and corresponding quantification of p-MAP4, p-MAP4 (S737), and p-MAP4(S760) in the skin tissues of *MAP4* KI mice. ***p < 0.001 versus the WT group. *n* = 5. **C** Representative bands and corresponding statistical analysis of LC3 in skin tissues. **p < 0.01 versus the WT group. *n* = 5. **D**, **E** Representative images and corresponding quantifications of autophagosomes. Yellow arrows pointed to autophagosomes. Scale bar = 0.5 μm. ***p < 0.001 versus WT group. *n* = 5. F Representative bands and corresponding statistical analysis of LC3 in primary MKs. **p < 0.01 versus the WT group. *n* = 5. **G**, **H** Representative bands and corresponding quantifications of autophagosomes in primary MKs. Yellow arrows pointed to autophagosomes/mitophagosomes. Scale bar = 0.5 μm. ***p < 0.001 versus WT group. *n* = 5. **I**, **J** Representative images and corresponding quantifications of GFP-LC3 positive keratinocytes. ***p < 0.001 versus WT group. *n* = 5. **K**, **L** Representative images and corresponding quantifications of autophagosome or autolysosome in keratinocytes measured by mRFP-GFP-LC3. Scale bar = 10 μm. *p < 0.05 and ***p < 0.001 versus WT group. *n* = 5. **M** Representative images of co-staining p-MAP4 with mitophagosomes. Scale bar = 10 μm. *n* = 5. **N**, **O** Representative bands and corresponding quantifications of p-MAP4 in keratinocytes treated by hypoxia with or without 30 min pre-treatment of 40 μM MDIVI-1. *p < 0.05 and ***p < 0.001 versus Con group. ^###^*p* < 0.001 versus Hypoxia (24 h) group. *n* = 5.

The above results suggested that p-MAP4 might activate autophagy in keratinocytes, but more efforts were required. With this purpose, the *MAP4* knockin (KI) mice were employed because they were an effective hyperphosphorylated MAP4 mice model, manifested by increased p-MAP4 at S737 and S760 sites (Fig. 2A, B, *p* < 0.05). Next, increased LC3-II/LC3-I ratio and enhanced mitophagosome number were then discovered in the skin tissue of *MAP4* KI mice (Fig. 2C–F, *p* < 0.05). More directly, primary MKs of WT or *MAP4* KI mice were isolated, and autophagy was measured once again. As shown in Fig. 2F–J, upregulated LC3-II/LC3-I ratio, augmented number of autophagosome represented by GFP-LC3 dots, and increased quantity of mitophagosome measured by TEM were also viewed in the primary MKs of *MAP4* KI mice (*p* < 0.05). These data demonstrated that p-MAP4 activated mitophagy in keratinocytes. Particularly, the autophagy flux induced by p-MAP4 was unobstructed, which was reflected by mRFP-GFP-LC3 staining (Fig. 2K, L, *p* < 0.05). And, p-MAP4 also gently alleviated hypoxia-impaired autophagy flux, which was demonstrated by nearly unchanged P62 expression but increased autolysosomes in keratinocytes co-treated by hypoxia and MAP4 (Glu) adenovirus (Fig. S1A–D). More importantly, p-MAP4 could be engulfed by mitophagosomes which was indicated by the co-localization of mitophagosomes with p-MAP4 in keratinocytes treated by hypoxia or from MAP4 KI mice (Fig. 2M). And, 40 μM MDIVI-1, a mitophagy inhibitor, could block the degradation of p-MAP4 induced by hypoxia (Fig. 2N, O). Together, p-MAP4 might undergo mitophagy-associated self-degradation in hypoxic keratinocytes.

### BH3 domain was the first requisite gateway of the mitophagy-associated self-degradation of p-MAP4 through functioning as an autophagy activator

**Fig. 3:**
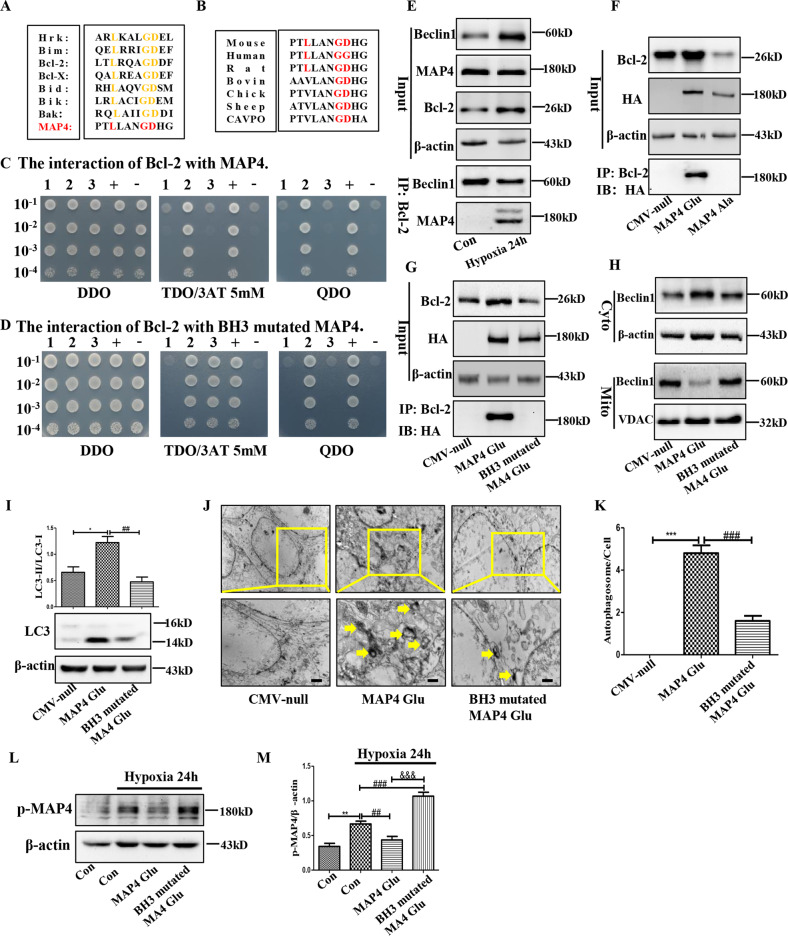
BH3 domain was the first requisite gateway of hypoxia-induced self-degradation of p-MAP4 through functioning as an autophagy activator. **A** The sequence alignment of MAP4 with classical BH3 domain. **B** The BH3 domain of MAP4 in different species. **C**, **D** Yeast two-hybrid (Y2H) screening systems was applied to investigate the direct interaction between Bcl-2 and MAP4 before or after the BH3 domain in MAP4 was mutated. **E** The Bcl-2/Beclin1 and Bcl-2/MAP4 interactions in HaCaT cells treated by hypoxia. *n* = 5. **F** The Bcl-2/MAP4 interaction in HaCaT cells treated by MAP4 (Glu) or MAP4 (Ala) adenovirus which was utilized to mimic the hyperphosphorylation or dephosphorylation condition of MAP4. *n* = 5. **G** The Bcl-2/MAP4 interaction in HaCaT cells treated by MAP4 (Glu) or BH3-mutated MAP4 (Glu) adenovirus. *n* = 5. **H** Representative bands of Beclin1 in cytoplasmic or mitochondrial proteins in HaCaT cells treated by MAP4 (Glu) or BH3-mutated MAP4 (Glu) adenovirus. *n* = 5. **I** Representative band and corresponding quantification of LC3 in HaCaT cells treated by MAP4 (Glu) or BH3-mutated MAP4 (Glu) adenovirus. *p < 0.05 versus CMV-null group. ^##^*p* < 0.01 versus MAP4 (Glu) group. *n* = 5. **J**, **K** Representative images and corresponding quantification of autophagosome in HaCaT cells treated by MAP4 (Glu) or BH3-mutated MAP4 (Glu) adenovirus. ***p < 0.001 versus CMV-null group. ^###^*p* < 0.001 versus MAP4 (Glu) group. *n* = 5. Yellow arrows point to autophagosomes. **L**, **M** Representative band and corresponding quantification of p-MAP4 in HaCaT cells treated by MAP4 (Glu) or BH3-mutated MAP4 (Glu) adenovirus. **p < 0.01 versus Con group. ^##^*p* < 0.01 versus Hypoxia (24 h) group. ^&&&^*p* < 0.001 versus Hypoxia (24 h) + MAP4 (Glu) group. *n* = 5.

The above data demonstrated p-MAP4 activated mitophagy, and the first key issue required to be solved was how p-MAP4 activated mitophagy. BH3 domain was a classical method to activate autophagy because it contributed to the release of Beclin1 from the Bcl-2/Beclin1 complex through competitively binding to Bcl-2 [10]. Therefore, the BH3 domain was the next focusing point. After sequence alignment, a potential and conserved BH3 domain was discovered in the N terminal of MAP4 (Fig. 3A, B). To further confirm this observation, yeast two-hybrid (Y2H) screening systems in vitro was utilized. As shown in Fig. 3C, direct interaction existed between MAP4 and Bcl-2. And, mutated MAP4, which inactivated the BH3 domain (knockout L83, G87, and D88), cleared the direct interaction between MAP4/Bcl-2 (Fig. 3D). Together, a BH3 domain was confirmed in MAP4.

Then, co-IP was conducted to investigate the interactions among MAP4/Bcl-2/Beclin1, and the result indicated that increased MAP4/Bcl-2 interaction and decreased Bcl-2/Beclin1 interaction were founded in hypoxic keratinocytes (Fig. 3E). The MAP4/Bcl-2 interaction in HaCaT cells transfected with MAP4 (Glu) or MAP4 (Ala) adenovirus was then measured, and the data showed MAP4/Bcl-2 interaction was phosphorylation-dependent (Fig. 3F), indicating that the BH3 domain was activated under the phosphorylated state of MAP4.

Next, BH3-mutated MAP4 (Glu) (L83/G87/D88A and S737/S760E) adenovirus which mimicked BH3 inactivation in p-MAP4 was constructed, and the adenovirus vanished MAP4/Bcl-2 interaction and downregulated the distribution of Beclin1 in cytoplasm (Fig. 3G, H). Next, BH3 inactivation decreased p-MAP4 elevated LC3-II/LC3-I ratio and autophagosome number (Fig. 3I-K, p < 0.05). Moreover, BH3 inactivation abolished hypoxia-induced self-degradation of p-MAP4 (Fig. 3L, M, *p* < 0.05). Together, these data demonstrated that BH3 activation was the first step of p-MAP4-activated mitophagy, and was also the first checkpoint of mitophagy-associated self-degradation of p-MAP4 in hypoxic keratinocytes.

### LIR (54-57) domain was the second requisite gateway of the mitophagy-associated self-degradation of p-MAP4 via functioning as a substrate recognition receptor

**Fig. 4:**
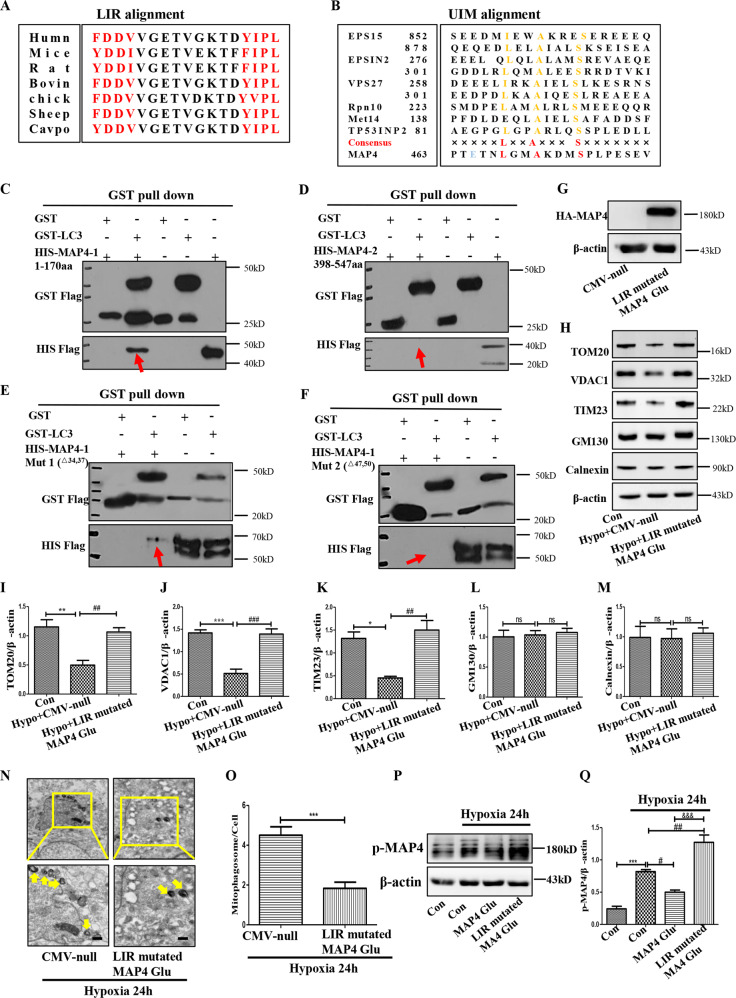
LIR (54-57) domain was the second requisite gateway of hypoxia-induced self-degradation of p-MAP4 via functioning as a substrate recognition receptor. **A**, **B** The potential LIR or UIM domains in MAP4 were discovered and aligned among different species. **C** The direct interaction of GST-LC3 and HIS-MAP4-1 (1–170 aa) measured by GST pull-down. *n* = 3. **D** The direct interaction of GST-LC3 and HIS-MAP4-2 (398–547 aa) detected by GST pull-down. *n* = 3. **E**, **F** The direct interaction of GST-LC3 with MAP4-1 Mut1^△34,37^ or MAP4-1 Mut2^△47,50^ detected by GST pull-down. *n* = 3. **G** The transfection efficiency of LIR mutated MAP4 (Glu) adenovirus in HaCaT cells. *n* = 3. **H**–**M** Representative bands and corresponding quantifications of TOM20, TIM23, VDAC1, GM130, and Calnexin in HaCaT cells treated by hypoxia with or without LIR mutated MAP4 (Glu) adenovirus. *p < 0.05, **p < 0.01, and ***p < 0.001 versus Con group, ^##^*p* < 0.01 and ^###^*p* < 0.001 versus Hypo + CMV-null group. *n* = 5. **N**, **O** Representative images and corresponding quantifications of autophagosomes in HaCaT cells treated by hypoxia with or without LIR mutated MAP4 (Glu) adenovirus. Scale bar = 0.5 μm. ***p < 0.001 versus CMV-null group. *n* = 3. Yellow arrows point to mitophagosomes. **P**, **Q** Representative band and corresponding quantification of p-MAP4 in HaCaT cells treated by hypoxia with MAP4 (Glu) or LIR mutated MAP4 (Glu) adenovirus. *p < 0.05 and ***p < 0.001 versus Con group. ^#^*p* < 0.05 and ^##^*p* < 0.01 versus Hypoxia (24 h) group. ^&&&^*p* < 0.001 versus Hypoxia (24 h) + MAP4 (Glu) group *n* = 5.

Another key issue of mitophagy-associated self-degradation of p-MAP4 was whether MAP4 could function as a substrate recognition receptor. LIR and UIM motifs have been verified to be the decisive structures of substrate recognition receptors [11, 12]. Luckily, two potential LIRs (34–37 aa and 47–50 aa) and a UIM (463–482 aa) were discovered in the MAP4 sequence, and the motifs were highly conservative among species (Fig.4A, B). Next, selective exogenous expression of WT MAP4 (MAP4-1:1–170 aa or MAP4-2:398-547 aa) was conducted for in vitro binding assays using GST-tagged LC3. This analysis indicated that MAP4 LIR truncation (1–170 aa), rather than UIM truncation (398-547 aa), mediated the direct interaction between MAP4 and LC3 (Fig. 4C, D). Considering two LIRs were contained in MAP4-1, MAP4-1 mut1 (LIR^△34,37^) and MAP4-1 mut2 (LIR^△47,50^) were then constructed, which facilitated the observation that MAP4 LIR (47–50 aa) was the only LIR responsible for MAP4/LC3 direct interaction (Fig. 4E, F). Together, MAP4 directly interacted with LC3 through its LIR (47-50 aa), endowing MAP4 with the capability to work as a substrate recognition receptor.

Given p-MAP4 translocated to mitochondria and activated mitophagy in hypoxic cells [4], whether MAP4 was a qualified mitophagy receptor was further investigated. To achieve this goal, LIR mutated MAP4 (Glu) adenovirus, which mimicked LIR inactivation in p-MAP4 was then constructed, and the results indicated that LIR inactivation abolished hypoxia-induced degradation of mitochondrial proteins and augmentation of mitophagosome number (Fig. 4G–O, *p* < 0.05). More importantly, LIR inactivation erased hypoxia-induced self-degradation of p-MAP4 (Fig. 4P, Q, *p* < 0.05). These data verified that LIR (47-50) made MAP4 a qualified mitophagy cargo receptor, and was the second checkpoint of the mitophagy-associated self-degradation of p-MAP4 in hypoxic keratinocytes.

### Inactivated BH3 domain blocked the proliferation and migration responses of keratinocytes to hypoxia

**Fig. 5:**
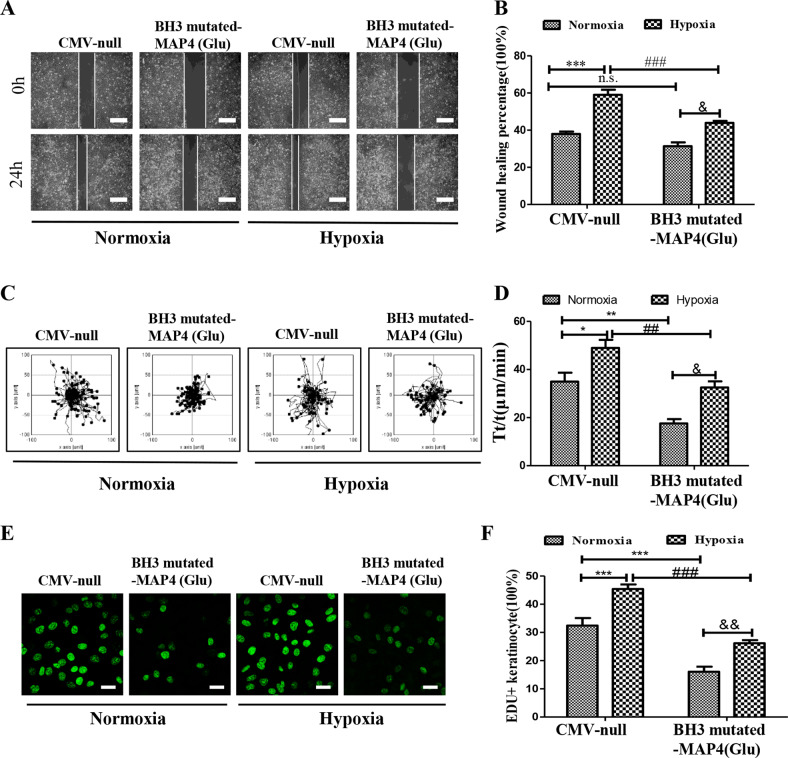
Inactivated BH3 domain blocked the proliferation and migration responses of keratinocytes to hypoxia. **A**, **B** Representative images and corresponding quantifications of scratch wound healing assays in HaCaT cells treated by hypoxia with or without BH3-mutated MAP4 (Glu) adenovirus. Scale bar = 100 μm.***p < 0.001 versus CMV-null group. ^###^*p* < 0.001 versus CMV-null + Hypoxia group. ^&^*p* < 0.05 versus BH3-mutated MAP4 (Glu) group. *n* = 5. **C**, **D** Representative images and corresponding quantifications of single-cell motility assays in HaCaT cells treated by hypoxia with or without BH3-mutated MAP4 (Glu) adenovirus. *p < 0.05 and **p < 0.01 versus CMV-null group. ^##^*p* < 0.01 versus CMV-null+Hypoxia group. ^&^*p* < 0.05 versus BH3-mutated MAP4 (Glu) group. *n* = 5. **E**, **F** Representative images and corresponding quantifications of EdU staining (Green) in HaCaT cells treated by hypoxia with or without BH3-mutated MAP4 (Glu) adenovirus. Scale bar = 50 μm. ***p < 0.001 versus CMV-null group. ^###^*p* < 0.001 versus CMV-null+Hypoxia group. ^&&^*p* < 0.05 versus BH3-mutated MAP4 (Glu) group. *n* = 5.

Next, the responses of keratinocytes which were overexpressed with BH3-mutated p-MAP4 to hypoxia, were then assayed. As shown in Fig. 5A, B, BH3-mutated MAP4 (Glu) adenovirus significantly inhibited hypoxia-induced wound healing (*p* < 0.05), as measured by scratch wound healing assay. Meanwhile, the velocity of single-cell movement stimulated by hypoxia was suppressed by BH3-mutated MAP4 (Glu) adenovirus in keratinocytes through employing a single-cell motility test (Fig. 5C, D, *p* < 0.05). Moreover, through EdU staining, the adenovirus downregulated hypoxia-induced proliferation of keratinocytes (Fig. 5E, F, *p* < 0.05). Together, BH3 inactivation inhibited the proliferation and migration responses of keratinocytes to hypoxia.

### Inactivated LIR (47–50) domain abolished the proliferation and migration responses of keratinocytes to hypoxia

**Fig. 6:**
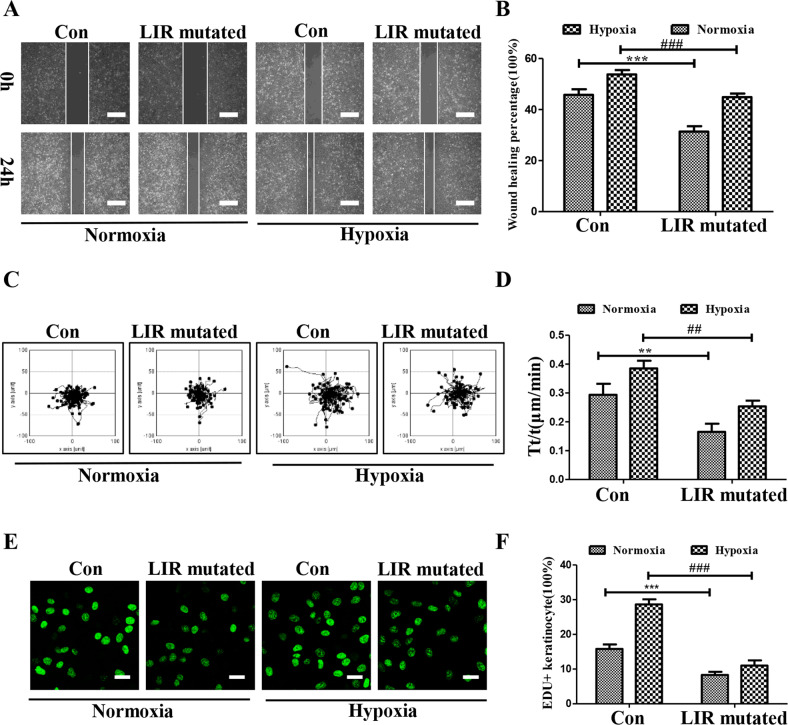
Inactivated LIR (47–50) domain abolished the proliferation and migration responses of keratinocytes to hypoxia. **A**, **B** Representative images and corresponding quantification of scratch wound healing assays in HaCaT cells treated by hypoxia with or without LIR mutated MAP4 (Glu) adenovirus. Scale bar = 100 μm.***p < 0.001 versus Con group. ^###^*p* < 0.001 versus Hypoxia group. *n* = 5. **C**, **D** Representative images and corresponding quantification of single-cell motility assays in HaCaT cells treated by hypoxia with or without LIR mutated MAP4 (Glu) adenovirus. **p < 0.01 versus Con group. ^##^*p* < 0.01 versus Hypoxia group. *n* = 5. **E**, **F** Representative images and corresponding quantifications of EdU staining (Green) in HaCaT cells treated by hypoxia with or without LIR mutated MAP4 (Glu) adenovirus. Scale bar = 50 μm. ***p < 0.001 versus Con group. ^###^*p* < 0.001 versus Hypoxia group. *n* = 5.

In this section, the influences of the LIR (47–50) domain on the proliferation and migration responses of keratinocytes to hypoxia were directly explored, and LIR (47–50) mutated MAP4 (Glu) adenovirus was used. As shown in Fig. 6A, B, LIR (47–50), mutated MAP4 (Glu) adenovirus showed an inhibitory effect on wound healing in scratch wound healing assay under hypoxia (*p* < 0.001). In addition, through single-cell mobility, LIR (47–50) mutated MAP4 (Glu) adenovirus was discovered to downregulate the elevated velocity of keratinocytes induced by hypoxia (Fig. 6C, D, *p* < 0.001). Furthermore, EdU staining was conducted, and the results demonstrated that LIR (47–50) mutated MAP4 (Glu) adenovirus and suppressed hypoxia augmented proliferation of keratinocytes (Fig. 6E, F, *p* < 0.05). Taking together, LIR inactivation disrupted the proliferation and migration responses of keratinocytes to hypoxia.

## Discussion

Previous studies have identified that p-MAP4 promoted wound healing under hypoxia through depolymerizing microtubules [2]. However, p-MAP4 should inhibit wound healing, for it translocated to and impairs mitochondria [4]. Thus, figuring out the outcome of p-MAP4 after it impaired mitochondria and how the outcome influenced wound healing were meaningful. With these purposes, current findings revealed that p-MAP4 experienced mitophagy-associated self-degradation, which was triggered by hypoxia in keratinocytes. And, this process was entirely dependent on the newly confirmed BH3 and LIR domains in the MAP4 sequence. As a result, mitophagy-associated self-degradation of p-MAP4 assured the migration and proliferation responses of keratinocytes to hypoxia. Together, our discoveries provided novel insights into investigating the effects of specific proteins on wound healing, and proved that the scavenging ability of autophagy might play a more central role than its other roles in mediating wound healing.

Previous article unveiled that both p-MAP4 and keratinocyte autophagy accelerated hypoxia-induced wound healing [2, 5]. And, the current data demonstrated that p-MAP4 augmented at 6 h and decreased at 24 h in hypoxic keratinocytes, but increased migration of keratinocytes presented at 24 h after hypoxia treatment. Therefore, the influences of p-MAP4 on the migration of keratinocytes might be indirect. Since the decrease in p-MAP4 expression was accompanied by a sustained increase in autophagy levels, whether p-MAP4 could be degraded by autophagy in hypoxic keratinocytes was speculated. However, autophagy flux induced by hypoxia was obstructed, and unsmoothed autophagy flux could not degrade p-MAP4, which has been proved through utilizing Baf A1. And, autophagy does consume p-MAP4, which has been confirmed by incubating keratinocytes with Rapa. During the process, p-MAP4 was discovered to activate autophagy. Further data showed that p-MAP4 stimulated mitophagy in keratinocytes, and mitophagy took a crucial position in hypoxia-induced degradation of p-MAP4. Thus, it was basically certain that p-MAP4 experienced mitophagy-associated self-degradation in keratinocytes, and this process was triggered by hypoxia.

To completely elucidate whether p-MAP4 could be self-degraded via mitophagy, the first key problem required to be solved was whether it activated mitophagy by itself. Beclin1 was a classical activator of autophagy and was also an important way of Bcl-2 homology domains (BH3 domains)-containing proteins induced autophagy activation [10]. In detail, BH3-containing proteins competitively bound to Bcl-2 and then released Beclin1 from the Bcl-2/Beclin1 complex, resulting in autophagy activation [10]. Based on these facts, a bold conjecture about whether MAP4 contained a classical BH3 domain was emerged. After careful alignment and detailed experiment, a typical BH3 domain in the N terminal of the MAP4 sequence was obtained and confirmed by yeast two-hybrid technique. To further prove whether the BH3 domain was the reason for p-MAP4-activated mitophagy, BH3 inactivated MAP4 (Glu) adenovirus was constructed. Exhaustive experiments showed that the BH3 domain was the mechanism of p-MAP4-induced autophagy activation. More importantly, inactivated BH3 domain abolished the self-degradation of p-MAP4 induced by hypoxia. Thus, the BH3 domain worked as the first checkpoint of hypoxia-triggered mitophagy-associated self-degradation of p-MAP4 through working as a mitophagy activator.

In addition, the confirmed BH3 domain made MAP4 a potential BH3-only protein family member. Even through, certain similarities and differences between MAP4 and traditional BH3-only proteins were existed. Firstly, most BH3-only proteins induced mitochondrial apoptosis [13], which was consistent with our previous findings about MAP4 [4]. However, their mechanisms were different, BH3-only proteins induced apoptosis was independent of mPTP and cytochrome c, but p-MAP4 was dependent [4, 14]. Secondly, the type of cell death induced by BH3-only proteins and MAP4 was different to some extent. Most BH3-only proteins stimulated apoptotic cell death [14], and p-MAP4 motivated pyroptosis [15]. Similar to MAP4, BNIP3, a well-known BH3-only protein, produced a different type of cell death which was described as necrotic cell death [14]. Therefore, MAP4 could work as a novel BH3-only protein with characteristics. Even so, to completely identify MAP4 as a BH3-only protein, more investigations should be conducted.

To implement the mitophagy-associated self-degradation, p-MAP4 should have the potential to work as a mitophagy substrate receptor. The LIR and UIM domains have been regarded as the structural basis of mitophagy substrate receptors [11, 12], and both of them have been discovered in the N terminal of MAP4. And, LIR (47–50 aa) has been confirmed to be the only reason for MAP4/LC3 interaction. This result lays a structural foundation for MAP4 to work as a mitophagy cargo receptor. In addition, p-MAP4 activated mitophagy in keratinocytes which has been first described in current research. These results conferred the potential of MAP4 as a mitophagy substrate receptor. And, the process of p-MAP4-mediated mitophagy could be described as follows. LC3-bound isolation membranes selectively recognized mitochondria which was tagged by MAP4, leading to mitochondrial incorporation, mitophagosome formation, and subsequent mitochondrial clearance. Furthermore, inactivated LIR (47–50) domain eliminated p-MAP4-induced mitophagy in keratinocytes and dispelled the self-degradation of p-MAP4 induced by hypoxia. Together, the LIR (47–50) functioned as the second checkpoint of hypoxia-triggered mitophagy-associated self-degradation of p-MAP4 through functioning as a mitophagy cargo receptor.

Based on the above evidences, MAP4 was thought to be a bran-new cargo receptor of mitophagy in eukaryotic cells because of its extensive distribution and conservative property [3, 16]. However, some differences existed between MAP4 and other well-known mitophagy cargo receptors. Traditionally, NIX and FUNDC1 were involved in hypoxia-induced mitophagy and were indispensable for the programmed elimination of mitochondria during reticulocyte maturation [17]. However, based on our findings, the mechanism of p-MAP4-induced mitophagy was different. Firstly, the expression of NIX or FUNDC1 was altered, whereas MAP4 remained unchanged. Besides, unlike FUNDC1, both NIX and MAP4 were distributed in other organelles under biological conditions and translocated to and impaired mitochondria under hypoxia [18, 19]. Moreover, the interaction of LC3/MAP4 was phosphorylation-dependent, which was consistent with LC3/Bnip3L interaction [20]. Therefore, MAP4 could work as a qualified mitophagy cargo receptor with specific characteristics. Even so, to completely identify MAP4 as a mitophagy cargo receptor, more efforts were required.

In the entire course of the present experiment, we discovered that every aspect of mitophagy-associated self-degradation of p-MAP4 was phosphorylation-dependent. This observation explained why hypoxia was the stimulator during the process because it phosphorylated MAP4 at S737 and S760 sites [3]. In line with our findings, post-translational modifications have been reported as stimulating factors under various pathological conditions. For example, phosphorylation impacted LC3/Bnip3L or LC3/Fundc1 interactions [20]. However, MAP4 was special and its particularity was that the phosphorylation sites (S737/760) and functional regions (LIR domain (Y47/L50) and BH3 domain (L83/G87/D88A)) were located in the C-terminal and N terminal, respectively. Consistently, Mariya et al. demonstrated that O-GlcNAcylation at the C-terminus of histone deacetylase 4 activated its N-terminus and then exhibited cardiac protection effects in diabetes models [21]. These evidences hinted that MAP4 might be a self-regulation protein, and post-translational modification was the mediator. Since MAP4 was divided into microtubule binding (MTB, the C-terminal) and projection (PJ, the N terminal) segments [3], its self-regulation could be understood as follows. Under biological conditions, MAP4 mainly showed its microtubule binding function through its MTB domain, and its PJ domain was inactivated. In contrast, under pathological conditions, post-translational modification at the MTB domain activated its PJ domain which contained BH3 and LIR motifs, and then participated in pathological responses, including mitophagy. Based on these facts, the mitophagy-associated self-degradation of MAP4 was affected by its self-regulation, and post-translational modification was the key.

Researchers have announced epidermal keratinocyte autophagy promotes wound healing through intervening crucial molecules of autophagy [5]. Given scavenging ability was the primary function of an autophagy-lysosome system [8], exploring the effects of autophagy on wound healing from its scavenging ability might be more significant. The current data has verified that MAP4 was a mitophagy-associated self-degradation protein. Therefore, MAP4 was a suitable molecule to elucidate the roles of autophagy on wound healing from the aspect of its scavenging ability. Our results showed that p-MAP4 with either LIR or BH3 inactivation delayed hypoxia and promoted wound healing. These facts indicated that p-MAP4 could be viewed as a toxic protein when its LIR or BH3 domain was inactivated. And, its toxicity could be cleared under specific conditions because wild-type p-MAP4 could undergo mitophagy-associated self-degradation. As a result, it guaranteed the migration and proliferation responses of keratinocytes to hypoxia. Together, scavenging toxic proteins might be the primary mechanism of autophagy-promoted wound healing.

Taken together, current research has identified p-MAP4 undergone mitophagy-associated self-degradation, which was triggered by hypoxia in keratinocytes. And, the self-degradation of p-MAP4 guaranteed the migration and proliferation responses of keratinocytes to hypoxia. From another aspect, scavenging toxic proteins might be the primary mechanism of autophagy-promoted wound healing. Therefore, our discoveries provided novel insights into investigating the effects of specific proteins on wound healing, and proved that the scavenging ability of autophagy might play a more central role than its other roles in mediating wound healing. Even so, more direct evidences were required.

## Materials and methods

### Ethical statement

All animal experiments were carried out according to the guidelines of the Care and Use of Laboratory Animals (NIH Pub. 8th edition, 2011). The Animal Experiment Ethics Committee of the Army Medical University (the Third Military Medical University) has approved the present study (approval number: AMUWEC2020413).

### Animal study

The *MAP4* (S667A, S737E, and S760E)-knockin (KI) mice were generated and bred as previously described [18]; WT (C57BL6/J) mice were purchased from the Animal Centre, Army Medical University (Third Military Medical University). Male mice, 6–8 weeks old and weighing 18–22 g, were used for the experiments. The mice were fed a standard rodent chow diet, allowed access to water ad libitum, and housed under 12 h light/dark cycles. Prior to the experiments, all animals were allowed to acclimate to the facility for one week. The animals were randomly divided into two groups, WT group and *MAP4*-KI group. Afterward, the mice were immediately euthanized, and their skin tissues were collected for transmission electron microscopy (TEM) or western blot (WB). Each animal-related test was repeated five times, and the sample size each time was 3. The sample size was estimated according to the ARRIVE Guidelines (Animal Research: Reporting of In Vivo Experiments).

### HaCaT cells study

Human epidermal keratinocyte cells (HaCaT) were the cell line routinely used [2, 9]. And, they were purchased from and identified by the cell Bank of the Chinese Academy of Sciences. The HaCaT cells were cultured in RPMI 1640 medium (HyClone, USA) supplemented with 10% fetal bovine serum (Gibco, USA), 100 U/ml penicillin, and 100 ug/ml streptomycin (Beyotime, China), and were incubated at 37 °C, 5% CO_2_, and 95% humidity. With different experimental purpose, the HaCaT cells were randomly treated with specific adenoviruses or chemical compounds. Each HaCaT cells-related test was repeated 5 times, and the sample size of each time was 3.

### Primary mouse epidermal keratinocytes (MKs) isolation and culture

Primary mouse epidermal keratinocytes (MKs) were isolated and cultured as previously described [2]. Briefly, the newborn (1–3 days) C57BL/6 or MAP4 KI mice were collected, and their skins were used for MKs isolation. Briefly, the separated skins were incubated with dispase treatment (4 °C) overnight and then were digested with 0.25% trypsin/0.04% EDTA solution (Invitrogen, USA) to obtain the MKs. The MKs were cultured in keratinocyte serum-free medium (K-SFM medium) (Gibco, USA) and were incubated at 37 °C, 5% CO_2_, and 95% humidity. With different experimental goals, the MKs suffered from specific treatments. Each MKs-related measurement was repeated five times, and the sample size for each time was 3.

### Hypoxia treatment

The HaCaT cells were exposed to hypoxic treatment when necessary, and the detailed information about hypoxia was as follows. A constant flow of nitrogen using a Forma Series II Water Jacket CO_2_ incubator (model:3131; Thermo Scientific) was used to create a hypoxic condition which was composed of 2% O_2_, 5% CO_2_, and 93% N_2_. The Forma Series II Water Jacket CO_2_ incubator could accurately maintain the desired temperature (37 °C) and O_2_ level. After hypoxia, the cells were further used to conduct a single-cell mobility assay, scratch wound healing assay or other experiments. Each hypoxia-related experiment was repeated five times, and three samples each time.

### HA-MAP4 (Ala), HA-MAP4 (Glu), HA-LIR-mutated MAP4 (Glu), and HA-BH3-mutated MAP4 (Glu) recombinant adenovirus construction and transduction

The HA-MAP4 (S737/760 A, Ala), HA-MAP4 (S737/760E, Glu), HA-LIR-mutated MAP4 Glu (F47/L50A and S737/S760E), and HA-BH3-mutated MAP4 Glu (L83/G87/D88A and S737/S760E, Glu) recombinant adenovirus were constructed by and purchased from Shanghai GeneChem, Co. Ltd. (Shanghai, CHN). The corresponding CMV-null adenoviruses were used as negative controls. Especially, MAP4 (Glu) and MAP4 (Ala) are used to mimic the hyperphosphorylation and dephosphorylation of MAP4. Each treatment related to these adenoviruses was repeated 5 times, and the sample size of each time was 3.

### Western blotting (WB) analysis

The protein samples of mouse skin or epidermal keratinocyte extracts were obtained using a total protein extraction kit (Beyotime, CHN). And then, the protein concentrations were detected using the Bradford Protein Quantification Kit (500-0205, Bio-Rad Laboratories). The samples were gone through the SDS-PAGE system and transferred to PVDF membranes (Millipore, USA). The membranes were incubated with specific primary antibodies overnight at 4 °C. The primary antibodies utilized were as follows. MAP4 (1:1000, Affinity, USA), p-MAP4 (1:1000, GL Biochem, CHN), p-MAP4 (S760) (1:1000, GL Biochem, CHN), p-MAP4 (S737) (1:1000, GL Biochem, CHN), LC3 (1:1000, sigma, USA), p62 (1:1000, sigma, USA), Beclin1 (1:1000, CST, USA), Bcl-2 (1:1000, CST, USA), VDAC1 (1:1000, Millipore, USA), Tomm20 (1:1000, Affinity, USA), TIM23 (1:1000, Affinity, USA), GM130 (1:1000, Affinity, USA), Calnexin (1:1000, Affinity, USA), HA-tag (1:1000, proteintech, USA) and β-Actin (1:1000, proteintech, USA). Then, corresponding secondary antibodies were used and the results were visualized using the ChemiDoc XRS System (Bio-Rad Laboratories). Each treatment was repeated 5 times, and the sample size for each time was 3.

### Immunofluorescence analysis

To detect autophagy/mitophagy status and cell proliferation in keratinocytes, the cells cultured on glass coverslips were transfected with GFP-LC3, Dsred-mito, or mRFP-GFP-LC3 adenovirus for 24 h or BeyoClick^TM^ EdU-488 for 2 h, and then the cells were fixed in 4% paraformaldehyde for 25 min which was followed by blocking with 10% goat serum for 1 h. Next, the cells were stained with DAPI for 1 h at room temperature. Finally, cells were imaged using confocal microscopy (Leica Microsystems, Wetzlar, Germany). Each treatment was repeated 5 times, and 3 samples each time.

### Transmission electron microscope (TEM)

The procedure of this part was according to the previous experiment [18]. After fixed in 2.5% glutaraldehyde, HaCaT cells, primary MKs, and skin tissues were successively subjected to dehydration, vibratome sliced and recut on a microtome and stained with uranyl acetate and lead citrate overnight. The sections were visualized through a TEM (JEM-1400 plus, Japan). Each treatment was repeated five times, and the sample size of each time was 3.

### Co-immunoprecipitation

To address the interaction between MAP4/Bcl-2 and Bcl-2/Beclin1, co-immunoprecipitation (co-IP) was conducted. Cells were lysed in RIPA buffer with protease inhibitor tablets. The MAP4, Bcl-2, or Beclin1 antibodies were incubated with cell lysate for 24 h at 4 °C, then the samples were precipitated with protein A/G-Sepharose (Santa Cruz, USA) overnight at 4 °C. The precipitates were washed five times with PBS at 0 °C and separated by SDS-PAGE and probed by rabbit anti-MAP4 (1:1000, Affinity, USA), anti-Bcl-2 (1:1000, CST, USA), and anti-Beclin1 (1:1000, CST, USA) using WB. Each treatment was repeated five times, and three samples each time.

### Yeast two-hybrid (Y2H) screening systems

Y2H systems were performed according to the Matchmaker Gold Yeast Two-Hybrid System User Manual. In brief, the entire *MAP4*, mutated *MAP4* with knocking out L83/G87/D88 and entire *Bcl-2* coding regions were successively amplified by PCR and respectively inserted in the pPR3-prey or pBT3-bait to generate a fusion with the GAL4 DNA binding domain (BD) or DNA activation domain (AD). The direct interaction of two proteins was investigated by co-transformation of the pBT3-STE-Bcl-2 and pPR3-N-MAP4 plasmids into the yeast strain NMY51, followed by dilution and selection of transformants on DDO, TDO or QDO plates at 30 °C for 3 days for growth selection. DDO, TDO, and QDO are used to detect bait plasmid toxicity and self-activation. Each treatment was repeated 3 times.

### Mitochondria fractions preparation

Mitochondrial fractions were prepared and validated from HaCaT cells according to the manual instruction of the Cell Fractionation Kit (Abcam, USA). Briefly, HaCaT cells were collected, counted, and diluted to 6.6 × 10^6^ cell/mL. Then, after being centrifuged at 500×*g* at 4 °C for 1 min, the supernatant was collected and again centrifuged at 10,000×*g* at 4 °C for 1 min. Then, the pellets were collected and resuspended, and after the same centrifugation process, the supernatants containing mitochondrial proteins were collected.

### Scratch wound healing assay

Monolayers of cells plated in six-well plates were wounded by a 10-μl plastic pipette tip after being incubated at 37 °C for 2 h and then rinsed with medium to remove cell debris [2]. The wound healing process was pictured with an inverted light microscope (Olympus, Japan) at 0 and 24 h. Cell migratory capacity was defined as the wound closure rate (%), which was analyzed using NIH ImageJ software. Each treatment was repeated five times, and the sample size of each time was 3.

### Single-cell motility assay and quantitative analysis

HaCaT or MKs at a density of 0.5 × 10^4^/cm^2^ were seeded into 24-well plates. Then, time-lapse imaging was performed with a Zeiss imaging system (Carl Zeiss Meditec, Jena, Germany), which maintained 37 °C and 5% CO_2_. The images were captured every 5 min for 3 h. Later, cell trajectories were analyzed by tracing the position of the cell nucleus at frame intervals of 10 min using NIH ImageJ software. The velocity (μm/min) of each cell was defined as the total length (μm) of the trajectory divided by time (min), which reflected cell motility. Each treatment was repeated five times, and the sample size of each time was 3.

### GST pull-down assay

GST pull-down assays were performed by GeneCreate Biological Engineering CO., Ltd (Wuhan, China). In brief, the *LC3*, *MAP4-1* (1–170 aa), *MAP4-2* (398–547 aa), *MAP4-1 LIR*^*Δ34,37*^, or *MAP4-1 LIR*^*Δ47,50*^ genes were first inserted into PGEX-6P-1 and pet-sumo vectors. The five recombinant plasmids were subsequently expressed in *Escherichia coli*, followed by the purification of GST-*LC3*, HIS-*MAP4-1*, HIS-*MAP4-2*, HIS-*MAP4-1 LIR*^*Δ34,37*^, and HIS-*MAP4-1 LIR*^*Δ47,50*^. The GST pull-down was conducted according to a previously published protocol [22], and measured via WB. Each treatment was repeated three times.

### Statistical analysis

All data were presented using the mean ± SEM. The statistical analysis was performed using SPSS, v. 22.0. The independent-sample *t*-test and the one-way analysis of variance were applied for comparisons between two groups or more than two groups, respectively. *P* < 0.05 was used as the threshold to define statistical significance. The error bars are generated according to SEM values.

## Supplementary information

Supplementary Figure Legends

Figure S1

Figure S2

Figure S3

Figure S4

Figure S5

Figure S6

## Data Availability

All data we used were contained in the manuscript.
