# Correction: Mitophagy associated self-degradation of phosphorylated MAP4 guarantees the migration and proliferation responses of keratinocytes to hypoxia

**DOI:** 10.1038/s41420-023-01498-8

**Published:** 2023-07-12

**Authors:** Yanhai Feng, Lingfei Li, Qiong Zhang, Yongqing He, Yao Huang, Junhui Zhang, Dongxia Zhang, Yuesheng Huang, Xia Lei, Jiongyu Hu, Gaoxing Luo

**Affiliations:** 1 Institute of Burn Research, Southwest Hospital, Third Military Medical University (Army Medical University), Chongqing, China; 2 State Key Laboratory of Trauma, Burns and Combined Injury, Southwest Hospital, Third Military Medical University (Army Medical University), Chongqing, China; 3 Army 953 Hospital, Shigatse Branch of Xinqiao Hospital, Third Military Medical University (Army Medical University), Shigatse, China; 4 Department of Dermatology, Daping Hospital, Third Military Medical University (Army Medical University), Chongqing, China; 5 Department of Geriatric Oncology, Department of Palliative care, Department of Clinical nutrition, Chongqing University Cancer Hospital, Chongqing, China; 6 Department of Wound Repair, Institute of Wound Repair and Regeneration Medicine, Southern University of Science and Technology Hospital, Southern University of Science and Technology School of Medicine, Shenzhen, China; 7 Endocrinology Department, Southwest Hospital, Third Military Medical University (Army Medical University), Chongqing, China

**Keywords:** Cell migration, Mitophagy

Correction to: *Cell Death Discovery* 10.1038/s41420-023-01465-3, published online 17 May 2023

In this article the author order was given erroneously. It should be read:

Yanhai Feng^1,2,3,4#^, Lingfei Li^4#^, Qiong Zhang^1,2#^, Yongqing He^4^, Yao Huang^1,2,3^, Junhui Zhang^5^, Dongxia Zhang^1,2^, Yuesheng Huang^6^, Xia Lei^4*^, Jiongyu Hu^2,7*^, Gaoxing Luo^1,2*^

Affiliations.

^1^Institute of Burn Research, Southwest Hospital, Third Military Medical University (Army Medical University), Chongqing, China;

^2^State Key Laboratory of Trauma, Burns and Combined Injury, Southwest Hospital, Third Military Medical University (Army Medical University), Chongqing, China;

^3^Army 953 Hospital, Shigatse Branch of Xinqiao Hospital, Third Military Medical University (Army Medical University), Shigatse, China;

^4^Department of Dermatology, Daping Hospital, Third Military Medical University (Army Medical University), Chongqing, China;

^5^Department of Geriatric Oncology, Department of Palliative care, Department of Clinical nutrition, Chongqing University Cancer Hospital, Chongqing, China;

^6^Department of Wound Repair, Institute of Wound Repair and Regeneration Medicine, Southern University of Science and Technology Hospital, Southern University of Science and Technology School of Medicine, Shenzhen, China;

^7^Endocrinology Department, Southwest Hospital, Third Military Medical University (Army Medical University), Chongqing, China;

The original article has been corrected.

