# Supplementary material for: Mitophagy associated self-degradation of phosphorylated MAP4 guarantees the migration and proliferation responses of keratinocytes to hypoxia
Source: Cell Death Discov. 2023 May 17;9:168. doi: 10.1038/s41420-023-01465-3 (PMC10192331; doi:10.1038/s41420-023-01465-3)
Supplement: Supplementary file 1 — Supplementary Figure Legends [file 41420_2023_1465_MOESM1_ESM.docx]

**Supplementary Materials**

Figure S1. Impaired autophagy flux induced by hypoxia was lightly alleviated by p-MAP4

(A-B). Representative band and corresponding quantification of P62 in keratinocytes treated by hypoxia with or without MAP4 (Glu) adenovirus. ^*^*p*<0.05 versus Con group, ns means not statistically significant. n = 5. (C-D). Representative images and corresponding quantifications of autophagy flux measured by mRFP-GFP-LC3 adenovirus in keratinocytes treated by hypoxia with or without MAP4 Glu adenovirus. Scale bar=10 μm. ^**^*p*<0.01 and ^***^*p*<0.001versus Hypoxia group. n=5.

Figure S2. The original bands of western blot in Figure 1. The bands with red frame were used.

Figure S3. The original bands of western blot in Figure 2. The bands with red frame were used.

Figure S4. The original bands of western blot in Figure 3. The bands with red frame were used.

Figure S5. The original bands of western blot in Figure 4. The bands with red frame were used.

Figure S6. The original bands of western blot in Figure S1. The bands with red frame were used.
