# Supplementary figures and images for: Mitophagy associated self-degradation of phosphorylated MAP4 guarantees the migration and proliferation responses of keratinocytes to hypoxia
Source: Cell Death Discov. 2023 May 17;9:168. doi: 10.1038/s41420-023-01465-3 (PMC10192331; doi:10.1038/s41420-023-01465-3)

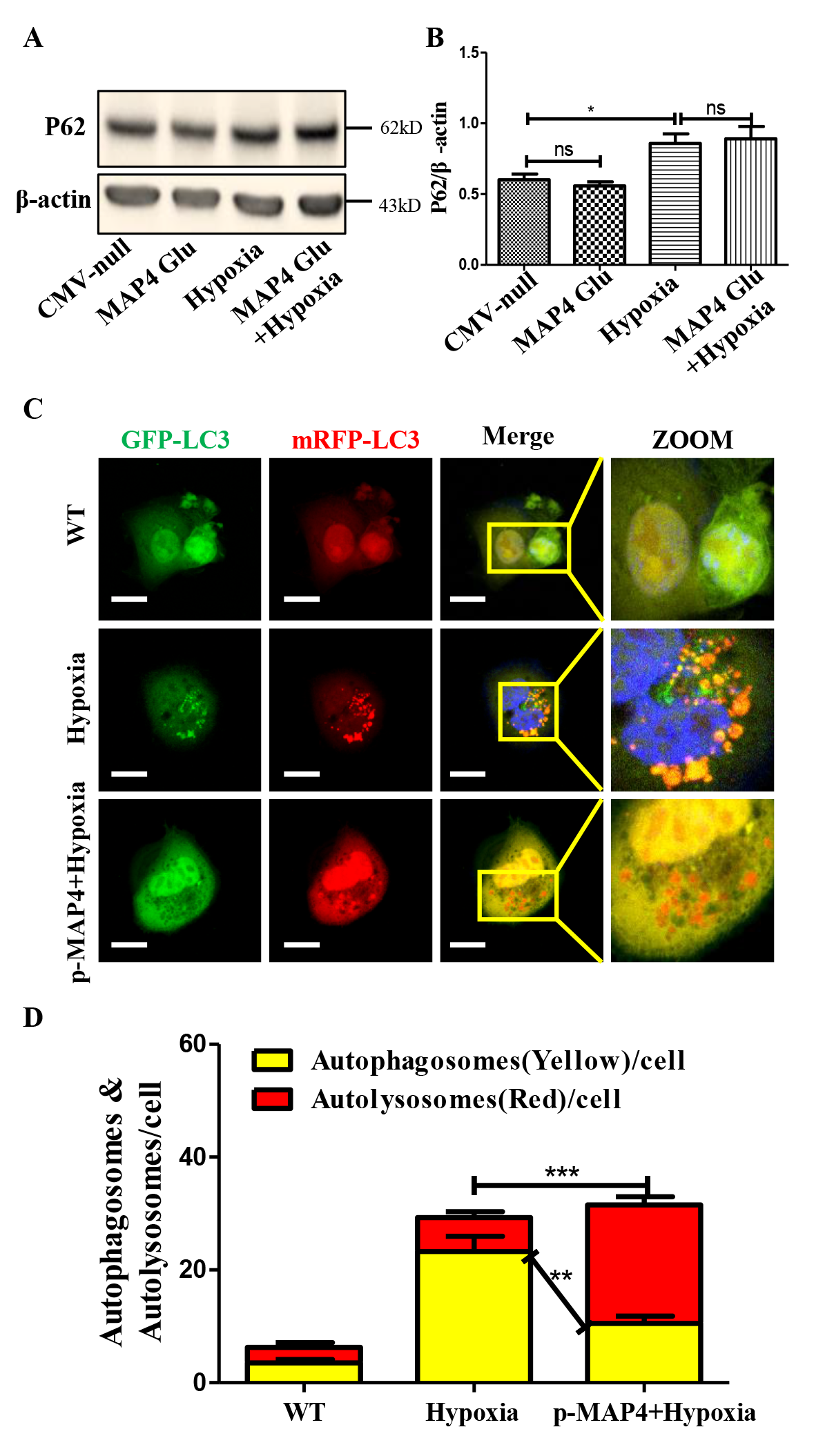

Supplement: Supplementary file 2 — Figure S1 [file 41420_2023_1465_MOESM2_ESM.tif]

Figure 1A

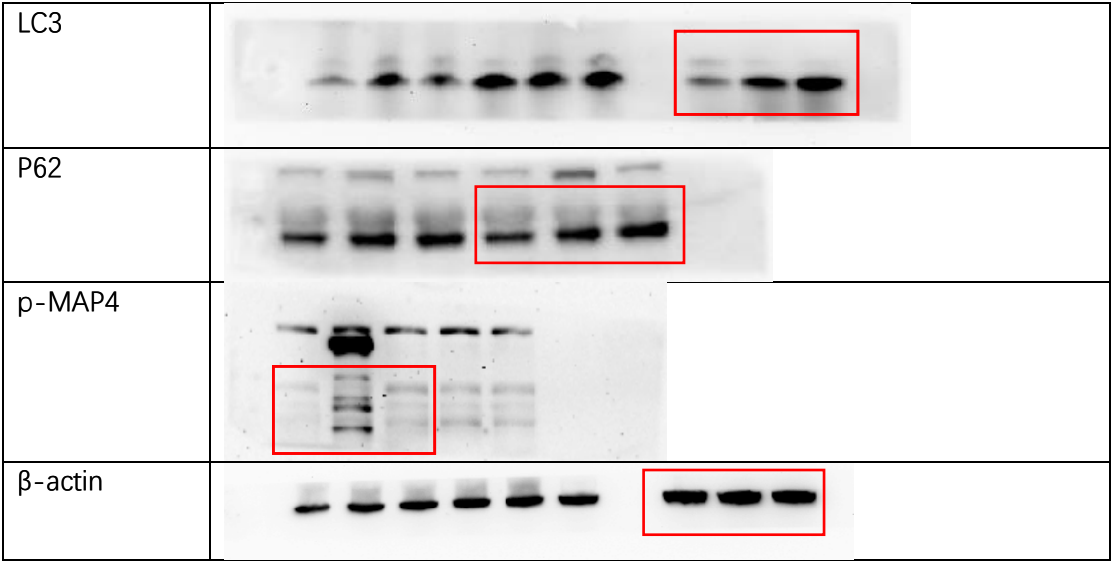

Figure 1M

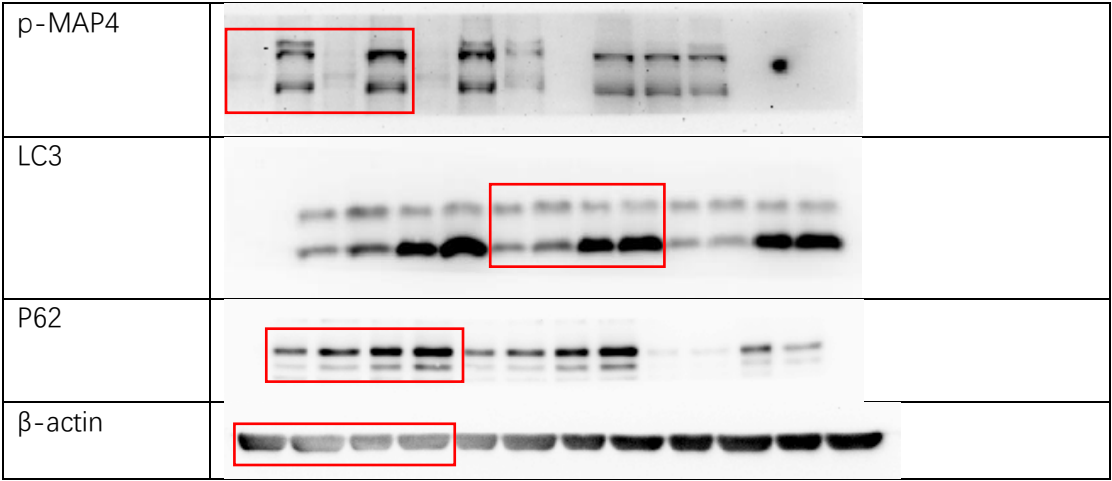

Figure 1Q

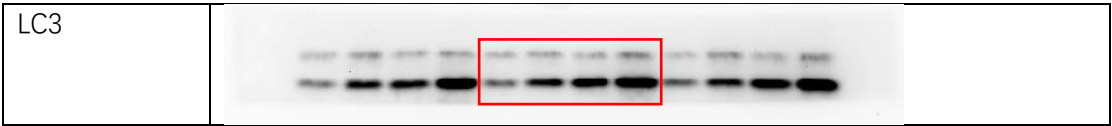

|                |                                                                                    |  |
|----------------|------------------------------------------------------------------------------------|--|
| P62            | 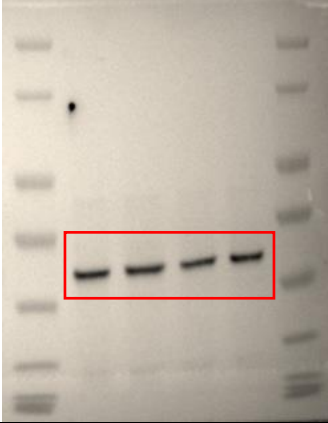  |  |
| p-MAP4         | 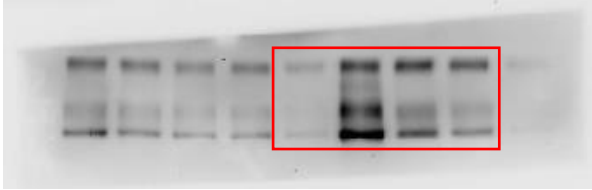 |  |
| $\beta$ -actin | 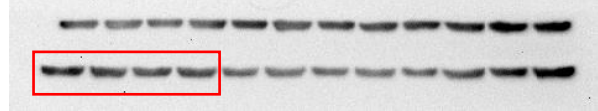 |  |

Supplement: Supplementary file 3 — Figure S2 [file 41420_2023_1465_MOESM3_ESM.pdf]

Figure 2A

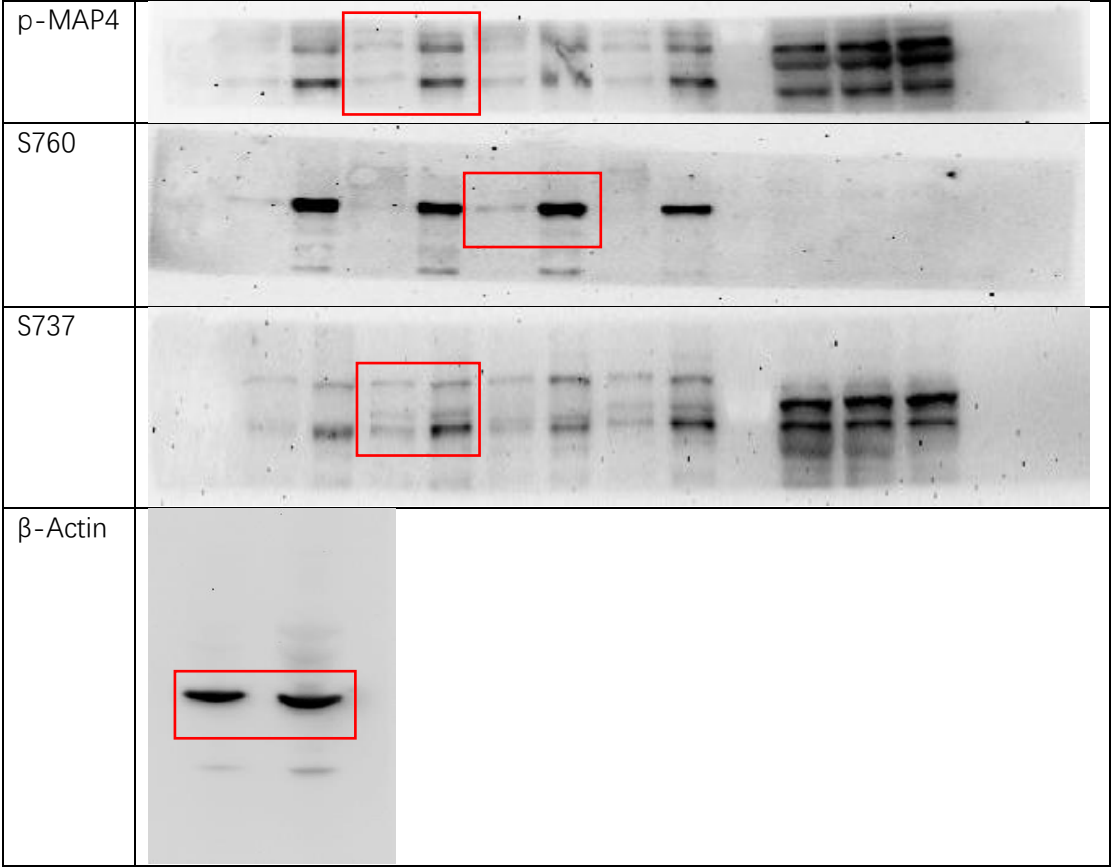

Figure 2C

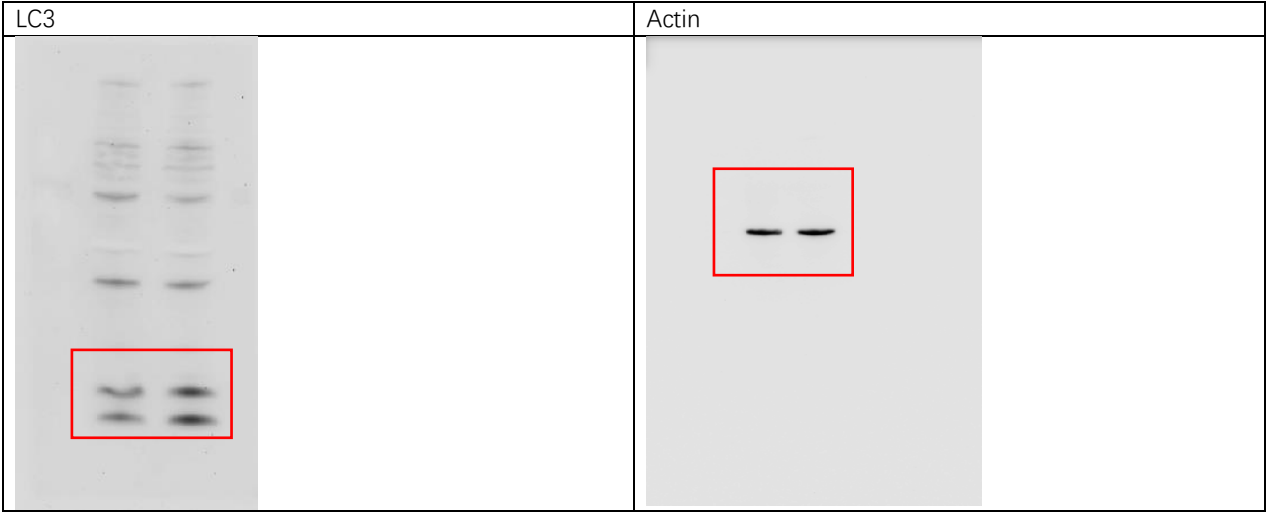

Figure 2F

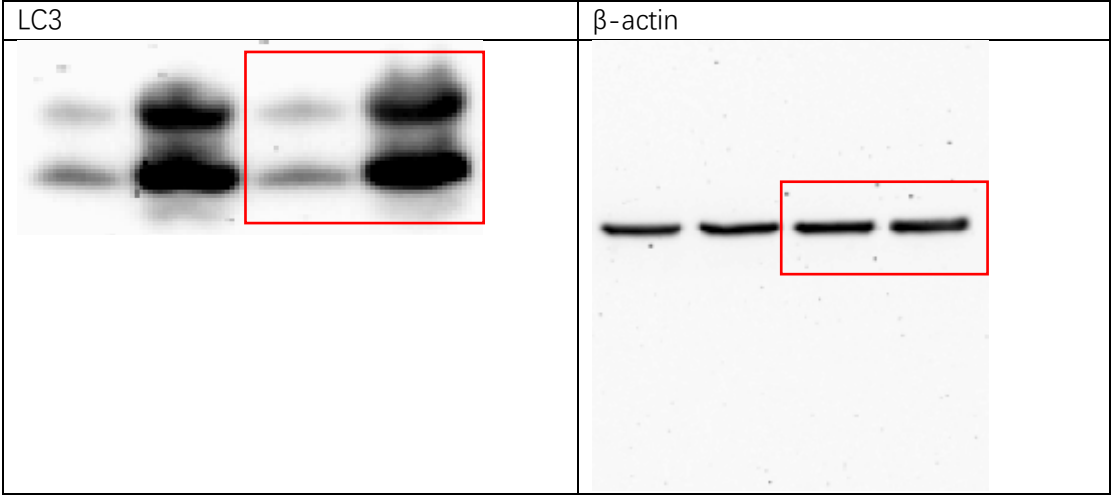

Figure 2L

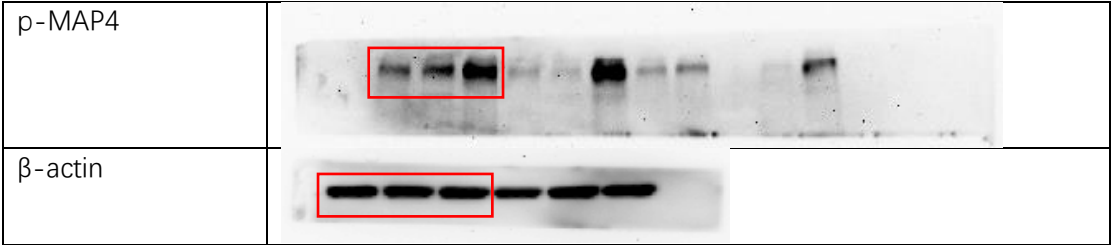

Supplement: Supplementary file 4 — Figure S3 [file 41420_2023_1465_MOESM4_ESM.pdf]

Figure 3E

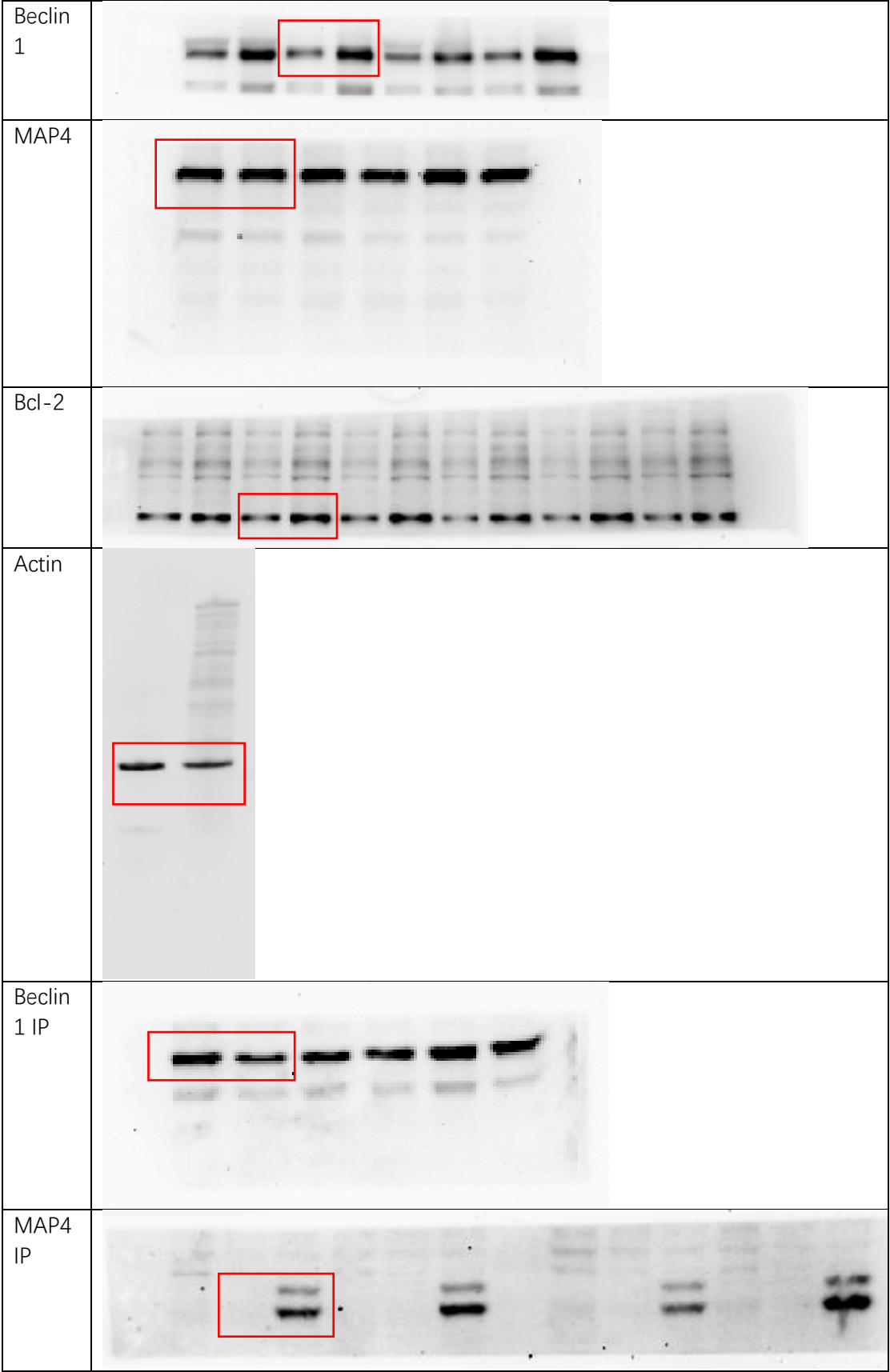

Figure 3F

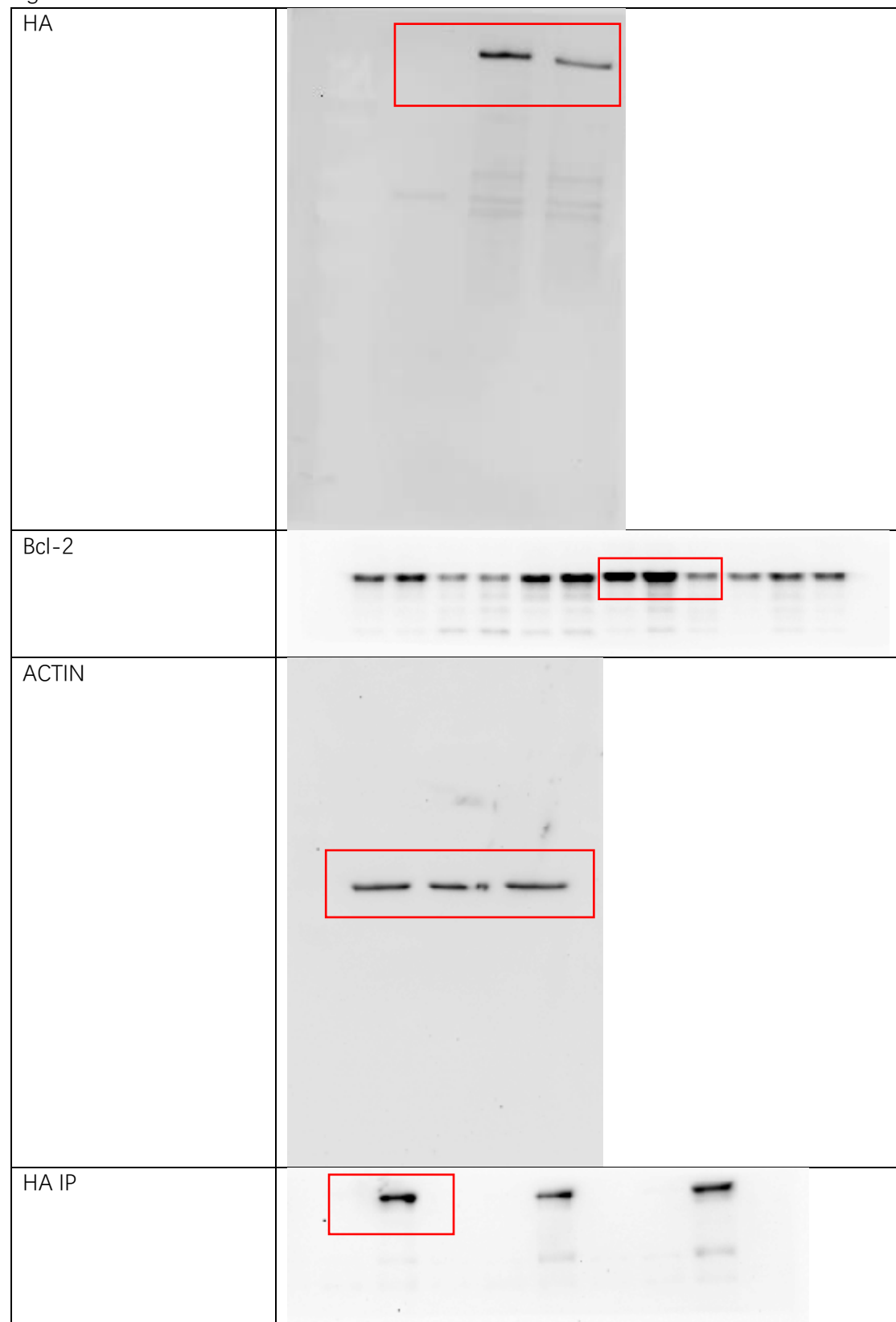

Figure 3G

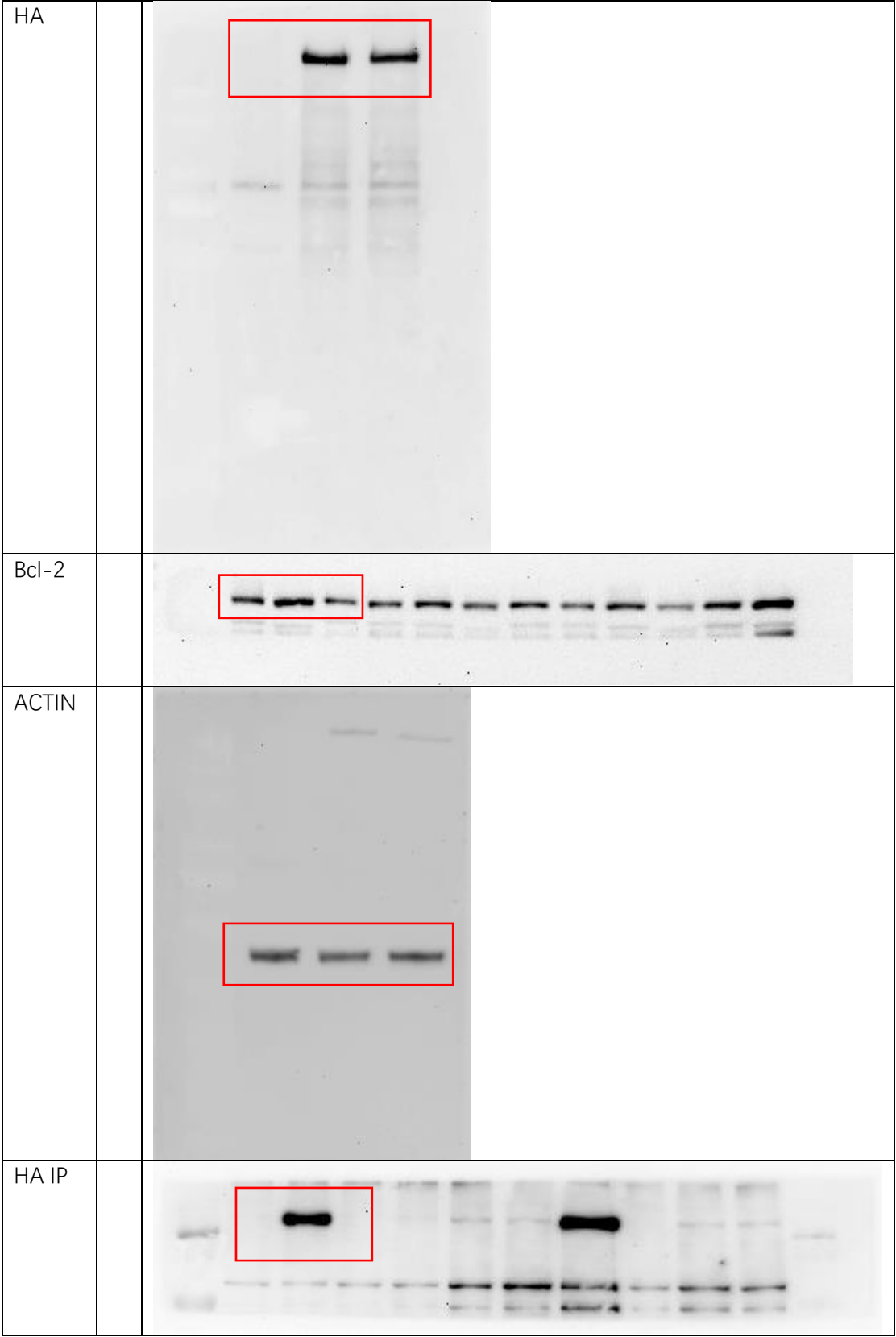

Figure 3H

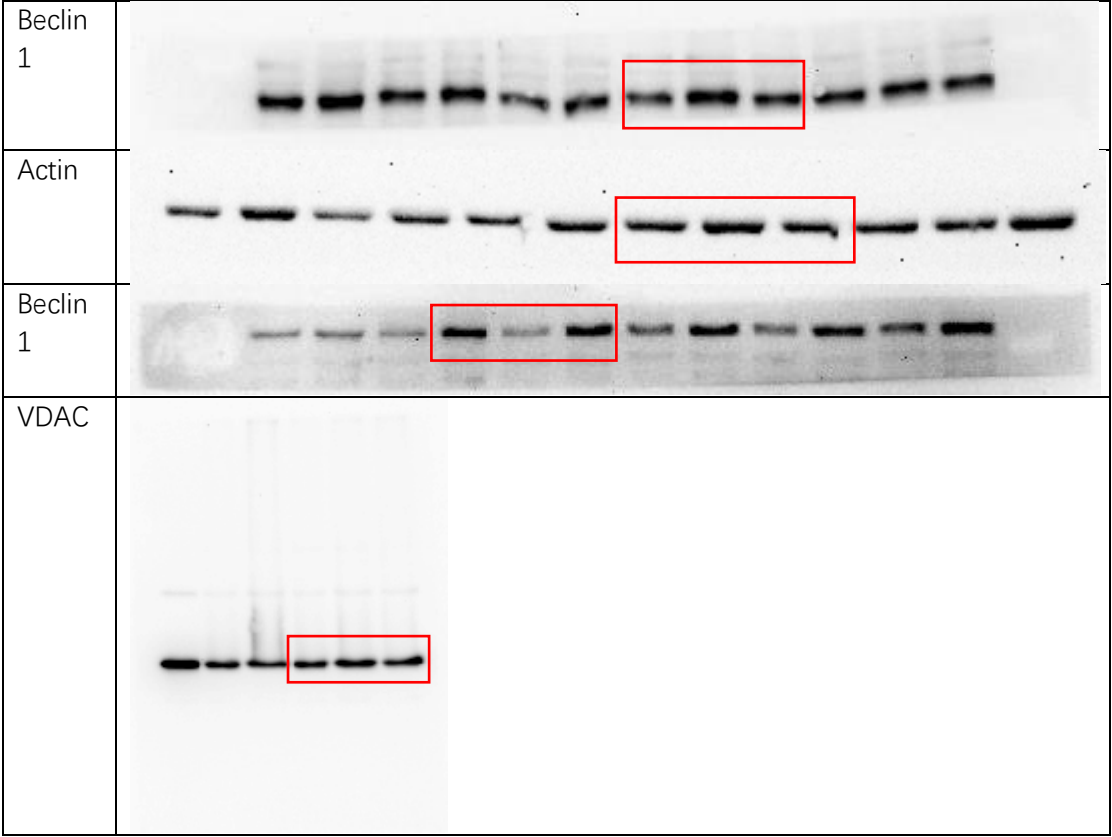

Figure 3L

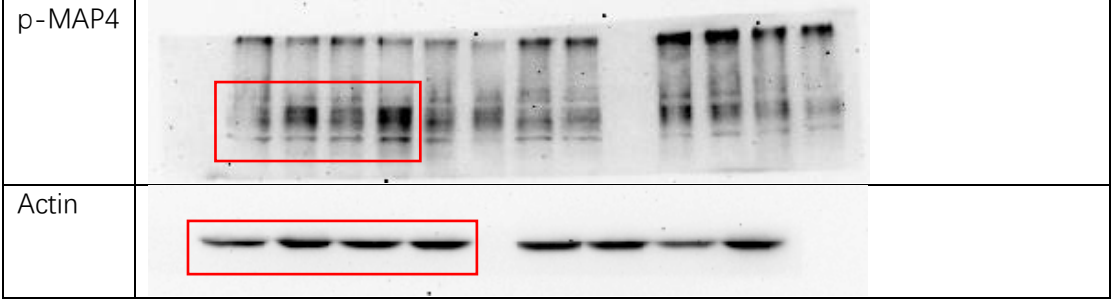

Supplement: Supplementary file 5 — Figure S4 [file 41420_2023_1465_MOESM5_ESM.pdf]

Figure 4G

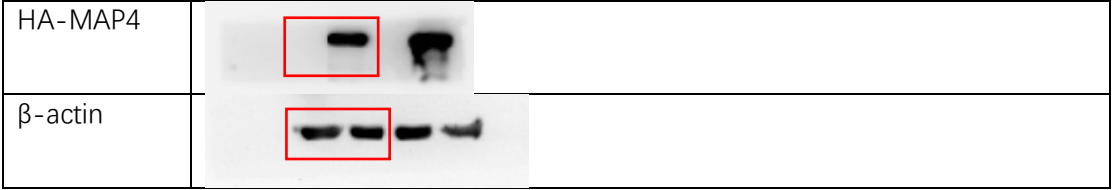

Figure 4H

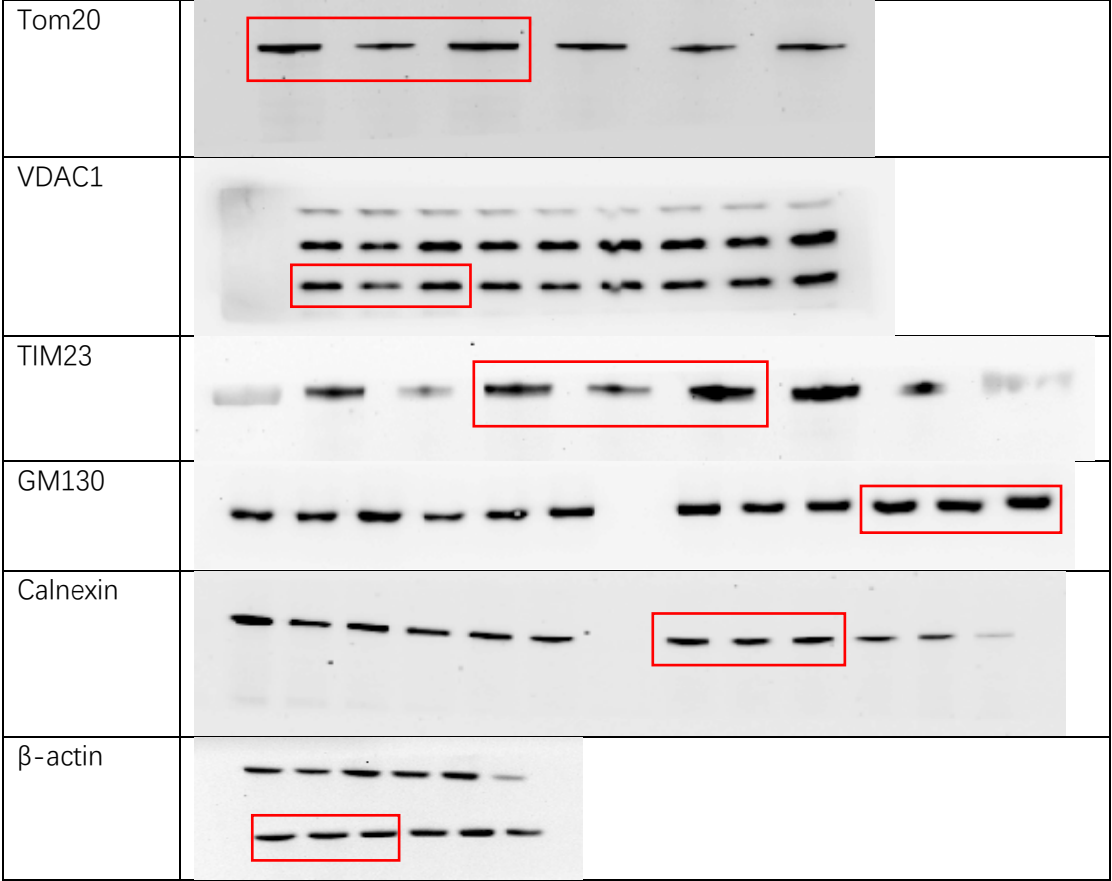

Figure 4P

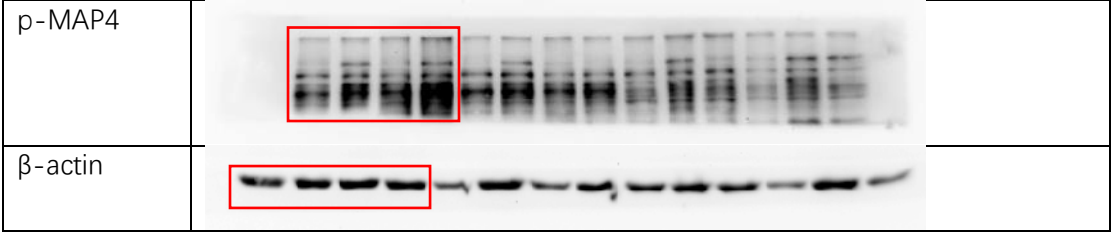

Supplement: Supplementary file 6 — Figure S5 [file 41420_2023_1465_MOESM6_ESM.pdf]

Figure S1A

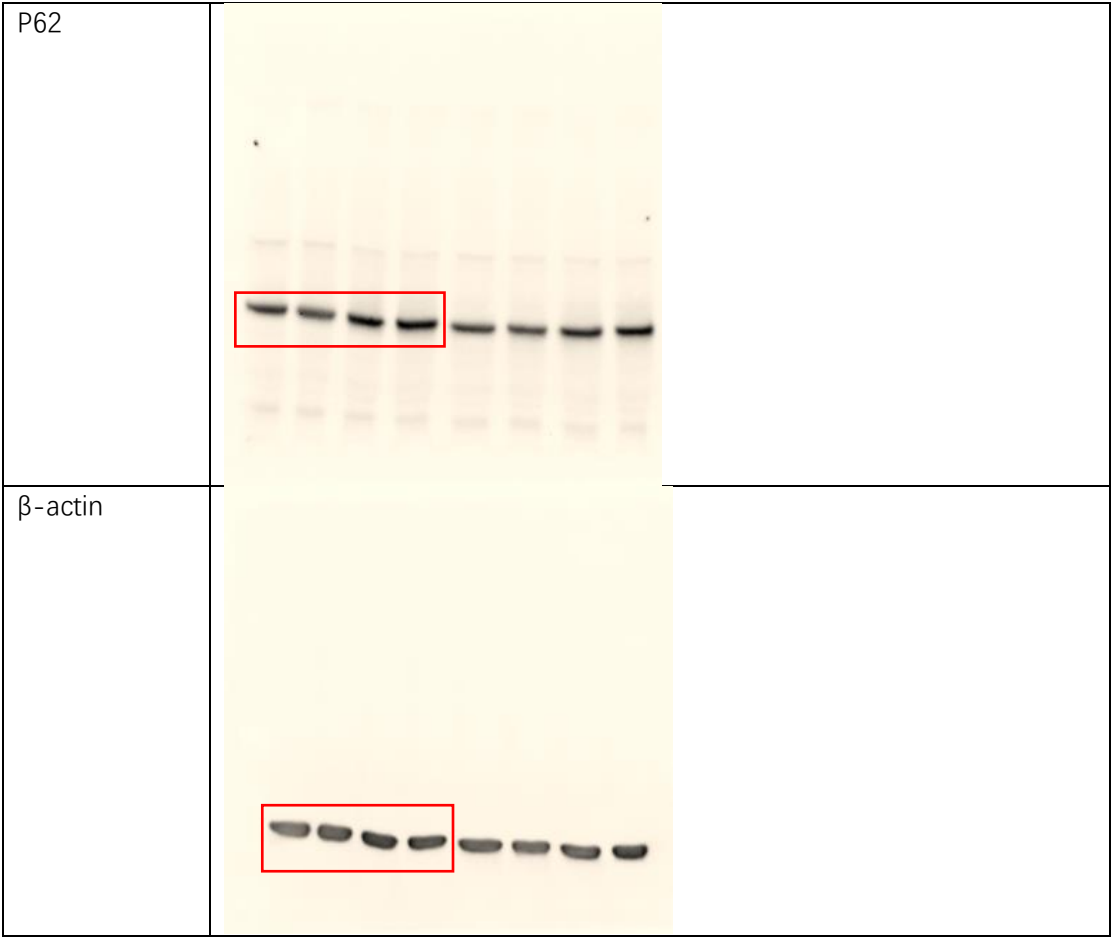

Supplement: Supplementary file 7 — Figure S6 [file 41420_2023_1465_MOESM7_ESM.pdf]
